# Senolytic compounds control a distinct fate of androgen receptor agonist- and antagonist-induced cellular senescent LNCaP prostate cancer cells

**DOI:** 10.1186/s13578-020-00422-2

**Published:** 2020-04-25

**Authors:** Thanakorn Pungsrinont, Malika Franziska Sutter, Maren C. C. M. Ertingshausen, Gopinath Lakshmana, Miriam Kokal, Amir Saeed Khan, Aria Baniahmad

**Affiliations:** 1 Institute of Human Genetics, Jena University Hospital, Am Klinikum 1, 07740 Jena, Germany; 2 Department of Hematology and Medical Oncology, Jena University Hospital, Jena, Germany; 3 Laboratory for Experimental Immunology of the Eye, Department of Ophthalmology, University Hospital Cologne, Cologne, Germany

**Keywords:** Prostate cancer, Cellular senescence, Senolytic compounds, HSP90 inhibitor, Bcl-2 family inhibitor, Akt inhibitor, Bipolar androgen therapy, Antiandrogen

## Abstract

**Background:**

The benefit of inducing cellular senescence as a tumor suppressive strategy remains questionable due to the senescence-associated secretory phenotype. Hence, studies and development of senolytic compounds that induce cell death in senescent cells have recently emerged. Senescent cells are hypothesized to exhibit different upregulated pro-survival/anti-apoptotic networks depending on the senescent inducers. This might limit the effect of a particular senolytic compound that targets rather only a specific pathway. Interestingly, cellular senescence in prostate cancer (PCa) cells can be induced by either androgen receptor (AR) agonists at supraphysiological androgen level (SAL) used in bipolar androgen therapy or by AR antagonists. This challenges to define ligand-specific senolytic compounds.

**Results:**

Here, we first induced cellular senescence by treating androgen-sensitive PCa LNCaP cells with either SAL or the AR antagonist Enzalutamide (ENZ). Subsequently, cells were incubated with the HSP90 inhibitor Ganetespib (GT), the Bcl-2 family inhibitor ABT263, or the Akt inhibitor MK2206 to analyze senolysis. GT and ABT263 are known senolytic compounds. We observed that GT exhibits senolytic activity specifically in SAL-pretreated PCa cells. Mechanistically, GT treatment results in reduction of AR, Akt, and phospho-S6 (p-S6) protein levels. Surprisingly, ABT263 lacks senolytic effect in both AR agonist- and antagonist-pretreated cells. ABT263 treatment does not affect AR, Akt, or S6 protein levels. Treatment with MK2206 does not reduce AR protein level and, as expected, potently inhibits Akt phosphorylation. However, ENZ-induced cellular senescent cells undergo apoptosis by MK2206, whereas SAL-treated cells are resistant. In line with this, we reveal that the pro-survival p-S6 level is higher in SAL-induced cellular senescent PCa cells compared to ENZ-treated cells. These data indicate a difference in the agonist- or antagonist-induced cellular senescence and suggest a novel role of MK2206 as a senolytic agent preferentially for AR antagonist-treated cells.

**Conclusion:**

Taken together, our data suggest that both AR agonist and antagonist induce cellular senescence but differentially upregulate a pro-survival signaling which preferentially sensitize androgen-sensitive PCa LNCaP cells to a specific senolytic compound.

## Background

For decades, prostate cancer (PCa) ranks the most diagnosed cancer and the second leading cause of cancer-related deaths of men in Western countries [1, 2]. It is well known that the androgen-activated androgen receptor (AR) plays a critical role for the growth of both normal and cancerous prostate [3]. Therefore, androgen deprivation therapy and inhibition of the AR-signaling by AR antagonists are the major forms of PCa hormone therapy. However, after a period of time, the cancer becomes resistant through adaptive responses of AR-signaling and activation of other signaling mechanisms [4–6]. Interestingly, supraphysiological androgen levels (SAL) are used in clinical trials so-called bipolar androgen therapy (BAT) as another approach to AR antagonists [7, 8].

We have previously described that cellular senescence can be induced by AR-signaling in PCa. On the one hand, several AR antagonists such as atraric acid, compound C28, or aminosteroids have been shown to induce cellular senescence in PCa cells [9–11]. On the other hand, induction of cellular senescence by supraphysiological level of AR agonists, the natural androgen dihydrotestosterone (DHT) or the less metabolized synthetic androgen methyltrienolone (R1881), were also described [12, 13]. Moreover, cellular senescence was induced in PCa samples from patients with prostatectomy ex vivo [13].

Cellular senescence is initially defined as an irreversible cell cycle arrest and has been proposed as one of the cancer inhibition strategies [14, 15]. However, the benefit of cellular senescence induction remains controversial. Although senescent cells can no longer divide, they are metabolically active and secrete cytokines, chemokines, growth factors, and proteinases known as the senescence-associated secretory phenotype (SASP). The SASP can mediate paracrine effects on neighboring non-senescent tumor cells and thus might act as a tumor promoter [16–19]. Therefore, induction of cellular senescence in cancer cells and elimination of these cells by senolytic compounds might be very useful to treat cancer.

Senolytic agents are molecular compounds that induce cell death in senescent cells [20]. Senescent cells might have an upregulated pro-survival/anti-apoptotic network, e.g. PI3K/Akt and/or Bcl-2/Bcl-xL pathway. Senolytic agents such as the HSP90 inhibitor Ganetespib (GT) and the Bcl-2 family inhibitor ABT263 have been described [21–23]. Notably, different senescence inducers might cause senescent cells to express a distinct upregulated pro-survival/anti-apoptotic pathway [24]. This might limit the ability of a particular senolytic agent that target specifically only one of these pathways.

In this study, we first induced cellular senescence in androgen-sensitive PCa LNCaP cells by treating with either AR agonist at SAL or antagonist, and subsequently analyzed the senolytic effects of GT, ABT263, and the highly selective allosteric Akt inhibitor MK2206. Our data suggest that the pro-survival Akt-S6 signaling is more active in AR agonist-induced cellular senescent cells than antagonist. Interestingly, treatment with GT or MK2206 after AR ligand-induced cellular senescence exhibits distinct senolytic activities depending on the type of AR ligand, agonist or antagonist, whereas ABT263 lacks senolytic activity.

## Results

### AR agonist and antagonist induce cellular senescence and suppress LNCaP cell growth

**Fig. 1:**
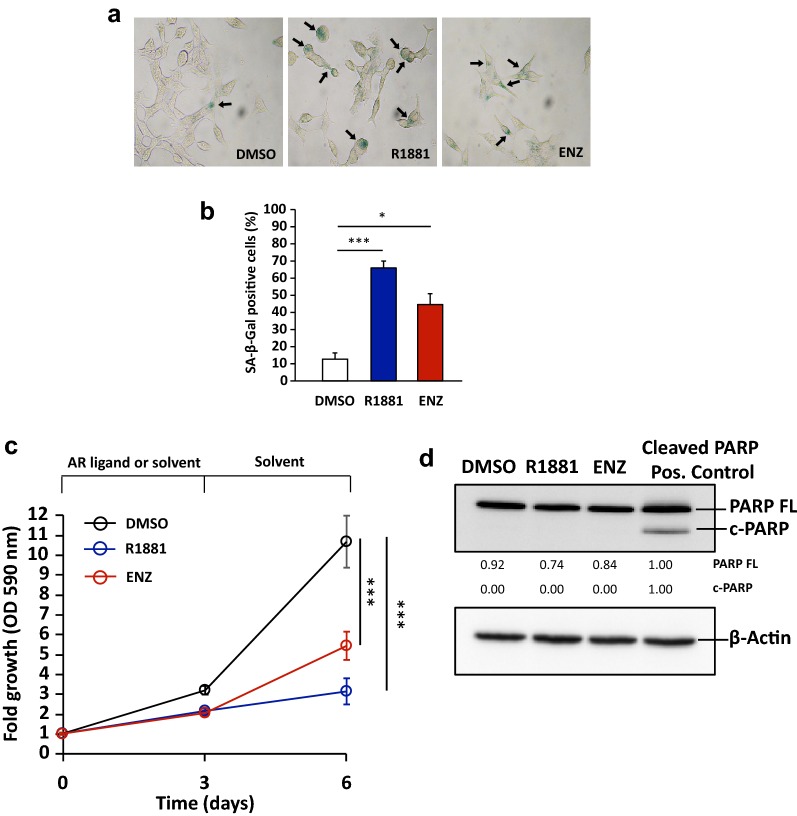
AR agonist and antagonist induce cellular senescence and suppress PCa LNCaP cell growth. LNCaP cells were treated for 72 h with 1 nM R1881 (agonist), 10 μM ENZ (antagonist), or 0.1% DMSO as solvent control. After that, the AR ligands were removed. Fresh medium with 0.1% DMSO was added and further incubated for additional 72 h. **a** Representative pictures of SA-β-Gal staining under light microscope. Arrows indicate SA-β-Gal positive stained cells. **b** Percentage of SA-β-Gal positive stained cells at 72 h of AR ligand treatment. Bar graphs are shown as mean ± SEM (n = 3). **c** Growth curves of LNCaP cells with indicated treatments and time points analysed by crystal violet staining and OD 590 nm measurement. Values obtained from day 0 were set arbitrarily as 1. Line graphs are shown as mean ± standard deviation (n = 5). **d** The protein extraction was performed after 72 h of AR ligand treatment. To detect cleaved PARP (c-PARP), protein extracted from LNCaP cells treated with 1 μM Akt inhibitor (MK2206) was loaded as positive control. Full-length PARP (PARP FL) and c-PARP were detected by Western blotting and normalized to β-Actin levels served as loading control. Upper and lower numbers indicate normalized PARP FL and c-PARP band intensities relative to positive control

In order to analyze the activity of senolytic agents in senescent LNCaP cells, first, cellular senescence was induced in the PCa cell line LNCaP cells by treating with AR agonist at SAL or antagonist for 72 h as described earlier [9, 13] (Fig. 1a, b). Thereafter, cells were treated with senolytic compounds to analyze cell growth, apoptosis, necroptosis and senescence markers. Since DHT can be rapidly metabolized and the metabolites can activate the estrogen receptor beta [25], the more stable and AR-specific compound R1881 was used as an AR agonist. As AR antagonist, the clinically approved Enzalutamide (ENZ) was used.

We have previously reported the capability of R1881 to induce cellular senescence in PCa cells [13]. Here, we show that the treatment with ENZ can also induce cellular senescence in LNCaP cells (Fig. 1a, b). In line with this, a significant increase of the senescence regulator *CDKN2A* (p16^INK4a^) mRNA was detected by ENZ treatment (Additional file 1: Fig. S1). Interestingly, a significant growth suppression of LNCaP cells after withdrawal of AR agonist or antagonist was observed (Fig. 1c). Moreover, we could not detect cleaved PARP, a marker for apoptosis, after AR ligand treatment (Fig. 1d), suggesting that AR ligands do not induce apoptosis but rather senescence in LNCaP cells. Thus, the data suggest that both AR agonist and antagonist induce cellular senescence leading to growth suppression of LNCaP cells.

### HSP90 inhibitor enhances apoptosis of AR agonist-induced cellular senescent LNCaP cells

Both the HSP90 inhibitor GT and the Bcl-2 family inhibitor ABT263 have been described as senolytic agents [21–23, 26]. Here, we show that both compounds inhibit LNCaP cell proliferation and induce apoptosis at higher concentrations (Additional file 1: Fig. S2). Notably, the growth inhibition and apoptosis induction by GT were observed after 48 h of treatment, whereas ABT263- or MK2206-induced apoptosis was detected after 24 h of treatment (Additional file 1: Fig. S2).

**Fig. 2:**
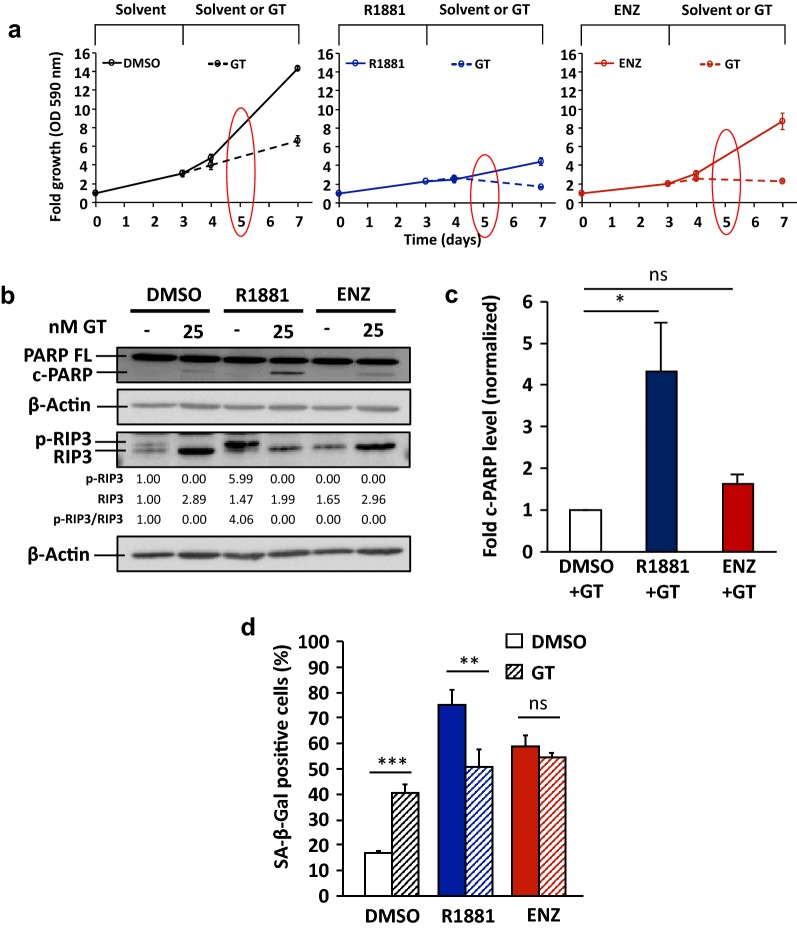
GT enhances apoptosis and reduces the proportion of SAL-induced cellular senescent PCa LNCaP cells. LNCaP cells were first treated for 72 h with 1 nM R1881, 10 μM ENZ, or 0.1% DMSO as solvent control. Thereafter, AR ligands were removed. Fresh medium with 0.1% DMSO or 25 nM GT was added and further incubated for the next 96 h. **a** Growth of LNCaP cells was analysed by crystal violet staining and OD 590 nm measurement. Values obtained from day 0 were set arbitrarily as 1. Line graphs are shown as mean ± standard deviation (n = 2). Red circles indicate the time point of protein extractions. **b** Protein extraction was performed after 48 h treatment with GT. Detection of full-length PARP (PARP FL), cleaved PARP (c-PARP), RIP3, and phosphorylated RIP3 (p-RIP3) was performed by Western blotting and normalized to β-Actin levels. Upper and middle numbers indicate normalized p-RIP3 and RIP3 band intensities relative to DMSO control. Lower numbers indicate the ratios of p-RIP3 versus RIP3 levels. **c** Quantification of fold c-PARP levels normalized to β-Actin from Western blotting data. Values obtained from DMSO + GT were set arbitrarily as 1. Bar graphs are shown as mean ± SEM (n = 3). **d** Percentage of SA-β-Gal positive stained cells after 48 h treatment with GT. Bar graphs are shown as mean ± standard deviation (n = 3)

To analyze senolytic activity of GT and ABT263 after cellular senescence was induced by SAL or ENZ treatment, 25 nM GT and 1 μM ABT263 were employed. Interestingly, GT treatment further suppressed cell growth after induction of cellular senescence by AR ligand (Fig. 2a). Detection of cleaved PARP indicates that GT treatment alone induces apoptosis and is more potent when cells are pretreated with SAL (Fig. 2b). Additionally, we analyzed necroptosis, another type of programmed cell death [27], by detecting the specific marker phospho-RIP3 (p-RIP3) (Fig. 2b and Additional file 1: Fig. S3). GT treatment with or without pretreatment with AR ligands reduces p-RIP3 level (Fig. 2b), suggesting that necroptosis is not the underlying mechanism of GT-induced cell death.

Prominently, GT treatment significantly enhanced cleaved PARP level after AR agonist-induced cellular senescence (Fig. 2b, c) indicating a senolytic activity of GT preferentially for SAL-treated cells. In line with this, we observed an enhanced detachment of cells and significant reduced percentage of SA-β-Gal positive cells by GT treatment after AR agonist-induced cellular senescence (Fig. 2d and Additional file 1: Fig. S4). Taken together, the results suggest that the known senolytic agent GT enhances apoptosis and further inhibits the growth of AR agonist-induced cellular senescent LNCaP cells.

### Treatment with Bcl-2 family inhibitor lacks senolytic activity in AR ligand-induced cellular senescent LNCaP cells

**Fig. 3:**
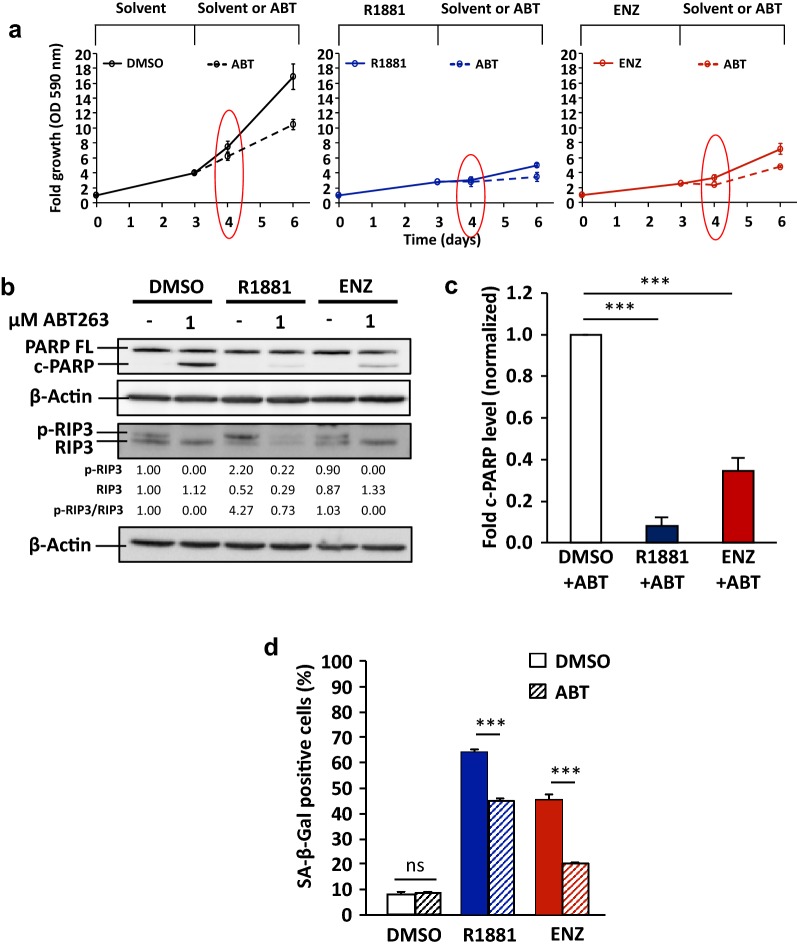
Treatment with ABT263 lacks senolytic effect in AR ligand-induced cellular senescent PCa LNCaP cells. LNCaP cells were first treated for 72 h with 1 nM R1881, 10 μM ENZ, or 0.1% DMSO as solvent control. After that, the AR ligands were removed. Fresh medium with 0.1% DMSO or 1 μM ABT263 was added and further incubated for additional 72 h. **a** Growth of LNCaP cells was analysed by crystal violet staining and OD 590 nm measurement. Values obtained from day 0 were set arbitrarily as 1. Line graphs are shown as mean ± standard deviation (n = 2). Red circles indicate the time point where protein extraction was performed. **b** The protein extraction was performed after 24 h treatment with ABT263. Detection of full-length PARP (PARP FL), cleaved PARP (c-PARP), RIP3, and phosphorylated RIP3 (p-RIP3) was performed by Western blotting and normalized to β-Actin levels. Upper and middle numbers indicate normalized p-RIP3 and RIP3 band intensities. Lower numbers indicate the ratios of p-RIP3 versus RIP3 levels. **c** Quantification of fold c-PARP levels normalized to β-Actin from Western blotting data. Values obtained from DMSO + ABT were set arbitrarily as 1. Bar graphs are shown as mean ± SEM (n = 3). **d** Percentage of SA-β-Gal positive stained cells after 24 h treatment with ABT263. Bar graphs are shown as mean ± SEM (n = 3)

Similar to GT treatment, LNCaP cell growth is suppressed by ABT263 (Fig. 3a), albeit less effective as GT. ABT263 treatment induces cleaved PARP and reduces p-RIP3 levels, indicating that ABT263 can induce apoptosis but not necroptosis (Fig. 3b). However, it is surprising that treatment with ABT263 after either AR agonist- or antagonist-induced cellular senescence exhibits significantly less cleaved PARP than the control-treated cells (Fig. 3b, c). Therefore, the data suggest that AR ligand-induced senescent LNCaP cells are more resistant to apoptosis induction by ABT263 than non-senescent cells.

Despite less detected cleaved PARP upon ABT263, we observed detachment of cells and reduced percentage of senescent/SA-β-Gal positive cells in AR ligand-pretreated cells (Fig. 3d and Additional file 1: Fig. S5). These data suggest that ABT263 causes cells to detach rather than to undergo apoptosis after AR agonist or antagonist treatment. Taken together, the results indicate that ABT263 might not have senolytic effect in AR ligand-induced cellular senescent PCa LNCaP cells.

### AR antagonist-induced cellular senescent LNCaP cells are sensitive to apoptosis induction by Akt inhibitor

It is known that activated Akt-signaling is one of the major cellular signaling pathways for cell survival in cancer [28]. MK2206 is a highly selective allosteric Akt inhibitor [29], which has been used in clinical studies against advanced solid tumors [30, 31]. Although MK2206 has also been used thoroughly for PCa cells in many studies [32–35], yet, the information of MK2206 activity for senescent cells is limited. Also, to our knowledge, it has not yet been described as a senolytic agent. Similar to the previous experiments, to analyze whether MK2206 mediates senolytic activity, cellular senescence was first induced by AR ligands and thereafter cells were treated with MK2206.

**Fig. 4:**
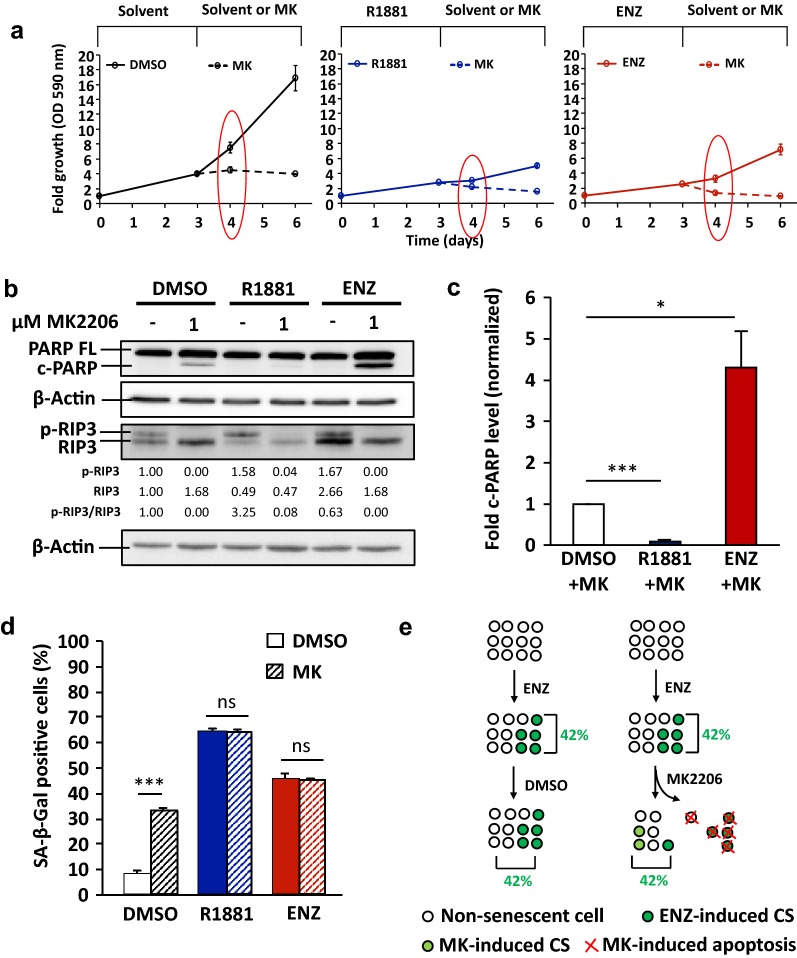
AR antagonist-induced cellular senescent PCa LNCaP cells are sensitive to apoptosis induction by MK2206. LNCaP cells were treated for 72 h with 1 nM R1881, 10 μM ENZ, or 0.1% DMSO as solvent control. After that, the AR ligands were removed. Fresh medium with 0.1% DMSO or 1 μM MK2206 was added and further incubated for additional 72 h. **a** Growth of LNCaP cells was analysed by crystal violet staining and OD 590 nm measurement. Values obtained from day 0 were set arbitrarily as 1. Line graphs are shown as mean ± standard deviation (n = 2). Red circles indicate the time point of protein extractions. **b** The protein extraction was performed after 24 h treatment with MK2206. Detection of full-length PARP (PARP FL), cleaved PARP (c-PARP), RIP3, and phosphorylated RIP3 (p-RIP3) proteins was performed by Western blotting and normalized to β-Actin levels. Upper and middle numbers indicate normalized p-RIP3 and RIP3 band intensities. Lower numbers indicate the ratios of p-RIP3 versus RIP3 levels. **c** Quantification of fold c-PARP levels normalized to β-Actin from Western blotting data. Values obtained from DMSO + MK were set arbitrarily as 1. Bar graphs are shown as mean ± SEM (n = 3). **d** Percentage of SA-β-Gal positive stained cells after 24 h treatment with MK2206. Bar graphs are shown as mean ± SEM (n = 3). **e** A schematic figure illustrates an unchanged/compensated percentage level of SA-β-Gal positive cells under MK2206 treatment of ENZ treated cell. Numbers represent the calculated percentage of SA-β-Gal positive cells

The results show that MK2206 inhibits cell proliferation in a concentration-dependent manner in the absence of AR ligand pretreatment (Additional file 1: Fig. S2). LNCaP cell growth is suppressed by MK2206 (Fig. 4a). In line with this, induced cleaved PARP but reduced p-RIP3 levels are observed at 24 h by MK2206 treatment with or without AR ligand pretreatment (Fig. 4b), suggesting that MK2206 induces rather apoptosis but not necroptosis.

Interestingly, detected cleaved PARP is significantly enhanced by MK2206 specifically after ENZ-induced cellular senescence (Fig. 4b, c). Furthermore, an enhanced detachment of cells by MK2206 after ENZ treatment was also observed (Additional file 1: Fig. S5). In contrast, AR agonist-induced cellular senescence exhibits significantly reduced cleaved PARP by MK2206 treatment indicating a protective mechanism against this Akt inhibitor by SAL (Fig. 4b, c). These results indicate that AR antagonist-treated cells are sensitive to MK2206 treatment and suggest MK2206 is an antagonist-specific senolytic compound, whereas agonist-treated cells are resistance.

An increased level of SA-β-Gal positive cells by MK2206 was observed in the control without pretreatment with AR ligand (Fig. 4d). This suggests that MK2206 is capable of inducing cellular senescence being in line with Xie et al. (2018) [26]. Surprisingly, by analyzing adherent cells, no reduction of SA-β-Gal positive cells by MK2206 after ENZ-induced cellular senescence was observed (Fig. 4d). We hypothesize that treatment with MK2206 on one hand triggered apoptosis and detachment of ENZ-induced cellular senescent cells and on the other hand induced cellular senescence in the remaining adherent cells (Fig. 4e). Hence, it resulted in an unchanged/compensated percentage level of SA-β-Gal positive cells.

Taken together, the data indicate that ENZ-induced cellular senescent LNCaP cells are sensitive to apoptosis induction by MK2206, whereas SAL-induced cellular senescent cells exhibit resistance. Therefore, the results suggest a novel role of MK2206 as a senolytic agent for AR antagonist-treated LNCaP cells.

### MK2206 and GT inhibit Akt-signaling, while ABT263 exhibits no effect

We hypothesized that AR agonist- and antagonist-induced cellular senescence upregulates different pro-survival/anti-apoptotic pathways. To address this, first the expression of Bcl-2 family members *BCL2*, *BCL2L2* and *BCL2L1* (encoding BCL-2, BCL-W, and BCL-XL) were analyzed. The results show that specifically agonist treatment significantly downregulates *BCL2* and upregulates *BCL2L1* mRNA expression (Additional file 1: Fig. S6), whereas no significant change is observed by antagonist treatment.

**Fig. 5:**
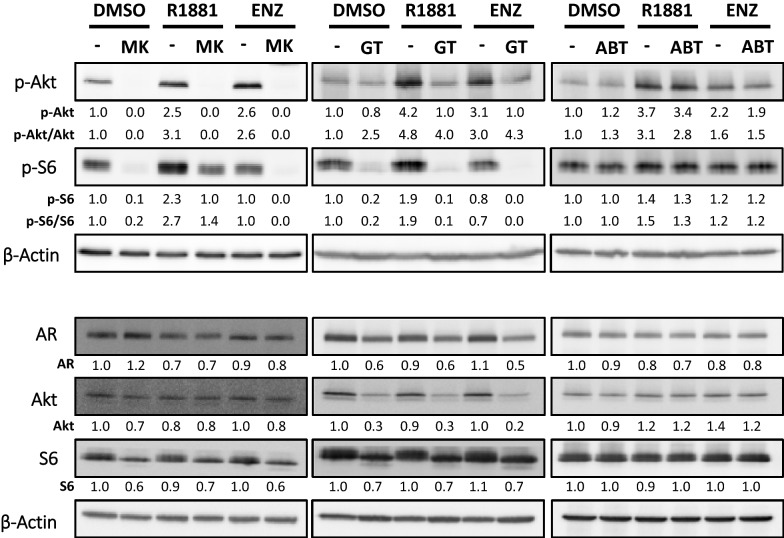
GT, ABT263, and MK2206 differentially regulate Akt-signaling and AR protein levels. LNCaP cells were treated for 72 h with 1 nM R1881, 10 μM ENZ, or 0.1% DMSO as solvent control. After that, the AR ligands were removed, replaced by fresh medium with 1 μM MK2206 (left panel), 25 nM GT (middle panel), 1 μM ABT263 (right panel), or 0.1% DMSO, and further incubated for additional 24 h (MK2206 and ABT263) or 48 h (GT). Protein extraction was conducted. Detection of AR, Akt, phospho-Akt (p-Akt), S6, and phospho-S6 (p-S6) was performed by Western blotting and normalized to β-Actin levels. For AR, pan-Akt and pan-S6, numbers indicate normalized band intensities relative to DMSO control. For p-Akt and p-S6, upper numbers indicate normalized band intensities relative to DMSO control, while lower numbers indicate ratios of phosphorylated versus total protein levels

Furthermore, phosphorylation of both Akt and S6, a downstream target of Akt as an indicator of Akt activity, were also analyzed (Fig. 5). Both phospho-Akt (p-Akt) and phospho-S6 (p-S6) protein levels are induced after SAL treatment. Interestingly, p-S6 level was not enhanced by ENZ suggesting a distinction between AR agonist- and antagonist-induced cellular senescence. This also indicates that Akt-S6 pathway is more active in AR agonist-treated cells (Fig. 5). Taken together, the expression of Bcl-2 family members, p-Akt, and p-S6 levels indicate that pro-survival/anti-apoptotic pathways are differentially regulated between AR agonist- and antagonist-induced cellular senescence.

Next, the effects of MK2206, GT, and ABT263 were analyzed on the pro-survival Akt-S6 pathway. After induction of cellular senescence by AR ligands, as expected, MK2206 potently reduces p-Akt levels (Fig. 5). Surprisingly, p-S6 levels, albeit decreased, remained high under MK2206 treatment in SAL-pretreated condition, suggesting that the activation of the downstream target of Akt, p-S6, is not fully blocked by MK2206 (Fig. 5). Hence, the data also indicate that the p-S6 level might be regulated in part by AR ligand, independent of Akt-phosphorylation. Notably, p-S6 itself, besides Akt, has also been implicated in cell survival [36–38]. MEFs with phospho-defective/unphosphorylatable S6 (S6^P−/−^) were more sensitive to apoptotic inducers than wild-type MEFs [37, 38]. This might explain our observation that SAL-induced cellular senescent cells exhibit resistance to MK2206.

Interestingly, GT treatment inhibits Akt-signaling as well (Fig. 5). In line with this, Akt, p-Akt, and p-S6 protein levels were downregulated by GT. Although GT does not reduce p-Akt level as strong as MK2206, p-S6 level is dramatically reduced by GT (Fig. 5). This suggests that the downstream Akt-signaling such as p-S6 is more effectively inhibited by GT compared to MK2206. Considering the possibility that the survival of SAL-treated cells relies on elevated p-S6 levels, reducing p-S6 levels by GT will potently induce apoptosis. This might explain the previous observation that GT enhances apoptosis after AR agonist-induced cellular senescence.

Unlike MK2206 and GT, the inhibitor of the Bcl-2 family ABT263 does not interfere with Akt-S6 pathway (Fig. 5). Both p-Akt and p-S6 levels remain similar with or without ABT263 treatment. Taken together, the results suggest that both MK2206 and GT distinctly inhibit Akt-signaling, while ABT263 seems not to affect this pathway.

### GT reduces AR protein expression, while ABT263 and MK2206 exhibit no effect

It is known that the AR is required for androgen-sensitive PCa cell survival. In line with this, AR knockdown mediates apoptosis in LNCaP cells [39]. Therefore, we analyzed AR protein level after GT, ABT263, and MK2206 treatment. Our data suggest that both MK2206 and ABT263 do not alter AR protein level (Fig. 5 and Additional file 1: Fig. S2). In contrast, GT reduces AR protein level regardless of the pretreatment with either AR agonist or antagonist (Fig. 5 and Additional file 1: Fig. S2). Since AR is a HSP90 client [40–42], the reduction of AR protein by GT is expected.

Taken together, our data suggest that the three inhibitors GT, ABT263, and MK2206 exhibit distinct mechanisms in AR ligand-induced cellular senescence. Mechanistically, GT treatment results in both reduction of AR protein level and inhibition of Akt-signaling. MK2206 does not reduce AR protein level but potently inhibits Akt phosphorylation, while ABT263 does neither affect AR protein nor Akt-signaling.

## Discussion

Cellular senescence has been firstly introduced as an anti-cancer purpose, however, the effects of senescent cells on the tumor microenvironment via SASP factors have emerged [16–19, 43]. SASP can mediate paracrine effects on neighboring non-senescent cells and might act as tumor promoter. Moreover, senescent cells are hypothesized to have upregulated pro-survival/anti-apoptotic networks [20]. Hence, it leads to development and characterization of senolytic agents to overcome specific pro-survival pathways in order to eliminate senescent cells.

In this study, we analyzed pro-survival pathways dependent on either AR agonist- or antagonist-induced cell senescence. First, we show that AR agonist R1881 at supraphysiological levels or the antagonist ENZ is capable of inducing cellular senescence but not apoptosis in the androgen-sensitive PCa LNCaP cells. We hypothesized that SAL or ENZ causes senescent cells to activate or upregulated distinct pro-survival/anti-apoptotic pathways. Our results indicate that the Akt-S6 pathway is more activated by AR agonist-induced cellular senescence compared to antagonist. Notably on the one hand, SAL induces phosphorylation of both Akt and S6. On the other hand, ENZ induces phosphorylation of Akt but not S6. Furthermore, in contrast to antagonist, AR agonist regulates the expression of Bcl-2 family members (*BCL2* and *BCL2L1*). These data suggest that the pro-survival/anti-apoptotic pathways are distinct between AR agonist- and antagonist-induced cellular senescent LNCaP cells. Therefore, a suitable senolytic compound for particular survival pathway must be identified.

Using inhibitors of these pathways indicated senolytic activity. Interestingly, our results reveal that treatment with Akt inhibitor MK2206 or HSP90 inhibitor GT exhibited distinct senolytic effects depending on the type of AR ligand, whereas no senolytic activity was observed for the Bcl-2 family inhibitor ABT263. In line with this, induction of the programmed cell death apoptosis but not necroptosis is observed as the underlying mechanism.

MK2206 is a highly selective allosteric Akt inhibitor [29] and to our knowledge, has not yet been described as a senolytic agent. Our data suggest that in contrast to AR agonist, the antagonist-induced cellular senescent PCa LNCaP cells are sensitive to apoptosis induction by MK2206. This demonstrates a novel role of MK2206 as senolytic compound for AR antagonist-treated LNCaP cells. In line with our findings, Pilling and Hwang (2019) showed that ENZ treatment induces phosphorylation of BAD (p-BAD) leading to inactivation of BAD, a pro-apoptotic factor [35]. Moreover, co-treatment of MK2206 and ENZ inhibit p-BAD, which was not observed with the treatment of MK2206 alone. The combination treatment resulted in an enhanced apoptosis when compared to MK2206 alone [35]. In case of antagonist-induced senescence, the Akt-BAD pathway might explain the antagonist-specific senolysis by MK2206.

In case of SAL-induced cellular senescence, we propose that the resistance to MK2206-mediated apoptosis is explained by the remaining high p-S6 levels despite Akt inhibition. Thus, we suggest that SAL treatment bypassed Akt and phosphorylated directly or indirectly S6. The p-S6 itself has also been implicated with cell survival [36–38]. The S6^P−/−^ mediates sensitivity to TRAIL-, etoposide-, or MG132-induced apoptosis in MEFs [37, 38], thus suggesting that p-S6 is a critical pro-survival factor. Therefore, we propose that despite treatment with the Akt inhibitor MK2206, SAL induces phosphorylation of S6 as an underlying mechanism for resistance against apoptosis by MK2206.

Further evidence supports the notion that AR agonist- and antagonist-induced cellular senescence address different intracellular factors. The HSP90 inhibitor GT selectively mediates high senolytic activity after AR agonist- but not antagonist-induced cellular senescence. In line with this, cleaved PARP induction was significantly enhanced by GT treatment after SAL-induced cellular senescence, indicating that AR agonist-treated LNCaP cells are sensitive to GT-induced apoptosis.

We hypothesize that the apoptosis induction by GT may rely on the synergistic reduction of AR protein level and inhibition of Akt-signaling. AR is required for androgen-sensitive PCa cell survival [39] and both AR and Akt are HSP90 clients [40–42, 44].

Interestingly, GT does not completely abolish the p-Akt level as observed with MK2206, however, p-S6 level is dramatically reduced by GT after SAL-induced cellular senescence. We hypothesize that the survival of AR agonist-treated LNCaP cells may also rely more on the upregulated/activated p-S6. Hence, AR agonist-induced cellular senescent cells are more sensitive to apoptosis when p-S6 is reduced.

ABT263 selectively induced apoptosis in senescent HUVECs and IMR90 cells, while no senolytic effect was observed in TW-37 cell types [45] suggesting a cell-type dependency. Surprisingly, our data suggest that ABT263 has no senolytic effect with either AR agonist- or antagonist-induced cellular senescence in LNCaP cells. Treatment with ABT263 in AR ligand-pretreated cells reveals significantly less cleaved PARP levels than the control-treated condition. Furthermore, treatment with ABT263 neither reduced AR protein nor inhibited Akt-signaling. The data further indicate that the AR ligand-induced cellular senescent LNCaP cells are more resistant to ABT263-mediated apoptosis than control.

We presume that it may depend very much on which pro-survival pathway is induced in senescent cells to use a specific senolytic compound. It is possible that between tumor types and within the heterogeneity of a tumor, a particular pro-survival pathway is activated by a senescence-inducing agent.

On the one hand, PCa therapy is currently relying on inhibition of the AR-signaling by AR antagonists as the major forms of hormone therapy. On the other hand, another option like BAT using SAL has recently been emerged. Either AR antagonist or SAL induces cellular senescence in PCa, including ex vivo in PCa patient samples [9, 13]. It is possible that the initial tumor growth reduction by therapy with AR antagonist or BAT is due to induction of cellular senescence. However, senescence might subsequently influence and stimulate neighboring tumor and non-tumor cells via SASP. Therefore, it might be useful to combine AR antagonist or BAT with senolytic compounds. Our data indicate that Akt inhibitor along with AR antagonist or HSP90 inhibitor along with SAL treatment may be beneficial therapeutic options.

## Conclusion

In conclusion, our data indicate that AR ligand-induced cellular senescent LNCaP cells exhibit different upregulated pro-survival pathways depending on the type of AR ligand used. Treatment with GT or MK2206 after AR ligand-induced cellular senescence exhibits distinct senolytic effects depending on the type of AR ligand and inhibitor, while ABT263 lacks senolytic effect. This suggests that senolytic activity of each senolytic compound can be limited when senescence is induced by different mechanisms. Thus, a suitable senolytic compound for a particular situation/treatment should be considered.

## Materials and methods

### Cell culture and treatments

LNCaP cells [46] were cultured in RPMI 1640 medium (Gibco Life Technologies) supplemented with 5% fetal calf serum (FCS), 100 U/ml penicillin, 100 µg/ml streptomycin, 1 mM sodium pyruvate, and 25 mM HEPES pH 7.5.

LNCaP cells were seeded in an appropriate amount for each experiment in cell culture plates. After 48 h of incubation, the cells were treated for 72 h with 1 nM R1881, 10 μM ENZ, or 0.1% DMSO as solvent control. After that, the medium including AR ligand was removed and refreshed by fresh medium with 25 nM GT, 1 μM ABT263, 1 μM MK2206, or 0.1% DMSO for the next 96 h. The cells were maintained in a 5% CO2, humidified atmosphere at 37 °C.

### Cellular senescence assays

The assays were performed with 6-well plates, and the cells were seeded at 25,000 cells per well. The staining and detection were performed as described previously [47–49]. The percentage of SA-β-Gal positive cells was calculated by counting at least 3 × 200 cells per well and at least 2 wells per treatment under a light microscope.

### Growth assays

The assays were performed with 6-well plates, and the cells were seeded at 20,000 cells per well. The growth assays were analyzed by crystal violet staining and OD 590 nm measurement, where the values obtained at day 0 were set arbitrarily as 1. The staining and detection were performed as described previously [48, 49].

### Antibodies and Western blotting

For protein extraction, the assays were performed with 10 cm cell culture plates, and the cells were seeded at 500,000 cells per dish for MK2206 experiments and at 120,000 cells per dish for GT and ABT263 experiments. The whole-cell lysate preparation was adapted from Esmaeili et al. (2016) [48, 49]. Briefly, the cells were lysed using NETN buffer (100 mM NaCl, 20 mM Tris/HCl pH 8.0, 1 mM EDTA, 1% NP‐40) supplemented with phosphatase inhibitors (5 mM NaF, 100 μM Na3VO4, 10 mM β‐Glycerophosphate) and followed by three cycles of freezing (in liquid nitrogen) and thawing (in a 37 °C water bath). The protein extracts were separated by SDS-PAGE. The primary antibodies used for immunodetection were Akt (Cell Signaling, 4685S), AR (Biogenex, 256M), β-Actin (Abcam, ab6276), phosphorylated Akt (S473) (Cell Signaling, 4058S), cleaved PARP (Cell Signaling, 9546S), RIP3 (Abcam, ab56164), S6 (Cell Signaling, 2217S), and phosphorylated S6 (S235/236) (Cell Signaling, 2211S). Horseradish peroxidase-conjugated anti-mouse IgG (Cell Signaling, 7076S) or anti-rabbit IgG (Cell Signaling, 7074S) were used as secondary antibodies. The detection was performed by ImageQuant^TM^ LAS 4000 (GE Healthcare Bio-Sciences AB).

To verify the detection of phosphorylated RIP3, the whole-cell lysate preparation was performed with NETN buffer with or without phosphatase inhibitors. The protein extracts were incubated at 37 °C for 3 h with or without alkaline phosphatase (FAST AP) (Thermo Scientific).

### Quantitative reverse transcription PCR (qRT-PCR)

The assays were performed with RNA isolated from 10 cm cell culture dishes, and the cells were seeded at 500,000 cells per dish. The total RNA extraction was performed using peqGOLD TriFast (Peqlab) according to the manufacturer’s protocol. Two-step qRT-PCR was performed as described previously [48, 49] with gene specific primers. *TBP* mRNA is served as the housekeeping gene for normalization. The primer sequences are listed as 5′ → 3′: *TBP*: fwd: GGCGTGTGAAGATAACCCAAGG, rev: CGCTGGAACTCGTCTCACT, *BCL2*: fwd: CATGTGTGTGGAGAGCGTCAA, rev: GCCGGTTCAGGTACTCAGTCA, *BCL2L1*: fwd: AACATCCCAGCTTCACATAACCCC, rev: GCGACCCCAGTTTACTCCATCC, *BCL2L2*: fwd: ACCCGTGAGATCCCTAACCTG, rev: CAGCTCCACAGACATAACCCT, *CDKN2A* (p14^ARF^): fwd: CCTGGAGGCGGCGAGAAC, rev: AGTAGCATCAGCACGAGGGC, *CDKN2A* (p16^INK4a^): fwd: CTTGCCTGGAAAGATACCG, rev: CCCTCCTCTTTCTTCCTCC, *CDKN1A*: fwd: TCGACTTTGTCACCGAGACACCAC, rev: CAGGTCCACATGGTCTTCCTCTG, *CDKN1B*: fwd: GGCCTCAGAAGACGTCAAAC, rev: ACAGGATGTCCATTCCATGA, *KLK3*: fwd: GAGGCTGGGAGTGCGAGAAG, rev: TTGTTCCTGATGCAGTGGGC.

### Statistical analysis

For statistical analysis, a two-tailed unpaired Student’s *t* test was performed using the GraphPad Prism 8.0 software, which were calculated from the mean, standard deviation, standard error of mean (SEM), and number of replicates (n). A 95% confidence interval (p-value (p) < 0.05) was considered as statistically significant (*) between two subject groups. A 99% confidence interval (p < 0.01) and a 99.9% confidence interval (p < 0.001) were indicated by two (**) and three stars (***), respectively. Western blotting analysis was performed at least for biological duplicates to shown regulation at protein levels.

## Supplementary information

**Additional file 1: Fig. S1.** Enzalutamide treatment significantly induces *CDKN2A* (p16^INK4a^) expression. **Fig. S2.** GT, ABT263, and MK2206 inhibit LNCaP cells proliferation through apoptosis induction in a concentration dependent manner. **Fig. S3.** p-RIP3 is dephosphorylated by alkaline phosphatase (FAST AP). **Fig. S4.** GT enhances detachment of cells after androgen-induced cellular senescence. **Fig. S5.** ABT263 and MK2206 induce LNCaP cell detachment. **Fig. S6.** Androgen regulates Bcl-2 family members transcription levels.

## Data Availability

All data generated and analyzed for this study are included in this published article.

## Abbreviations

AR: Androgen receptor
BAT: Bipolar androgen therapy
DHT: Dihydrotestosterone
ENZ: Enzalutamide
GT: Ganetespib
HSP90: Heat shock protein 90
PCa: Prostate cancer
R1881: Methyltrienolone
SA-β-Gal: Senescence-associated β-galactosidase
SAL: Supraphysiological androgen level
SASP: Senescence-associated secretory phenotype
