# Supplementary material for: Senolytic compounds control a distinct fate of androgen receptor agonist- and antagonist-induced cellular senescent LNCaP prostate cancer cells
Source: Cell Biosci. 2020 Apr 25;10:59. doi: 10.1186/s13578-020-00422-2 (PMC7183592; doi:10.1186/s13578-020-00422-2)
Supplement: Supplementary file 1 — Additional file 1: Fig. S1. Enzalutamide treatment significantly induces CDKN2A (p16INK4a) expression. Fig. S2. GT, ABT263, and MK2206 inhibit LNCaP cells proliferation through apoptosis induction in a concentration dependent manner. Fig. S3. p-RIP3 is dephosphorylated by alkaline phosphatase (FAST AP). Fig. S4. GT enhances detachment of cells after androgen-induced cellular senescence. Fig. S5. ABT263 and MK2206 induce LNCaP cell detachment. Fig. S6. Androgen regulates Bcl-2 family members transcription levels. [file 13578_2020_422_MOESM1_ESM.pdf]

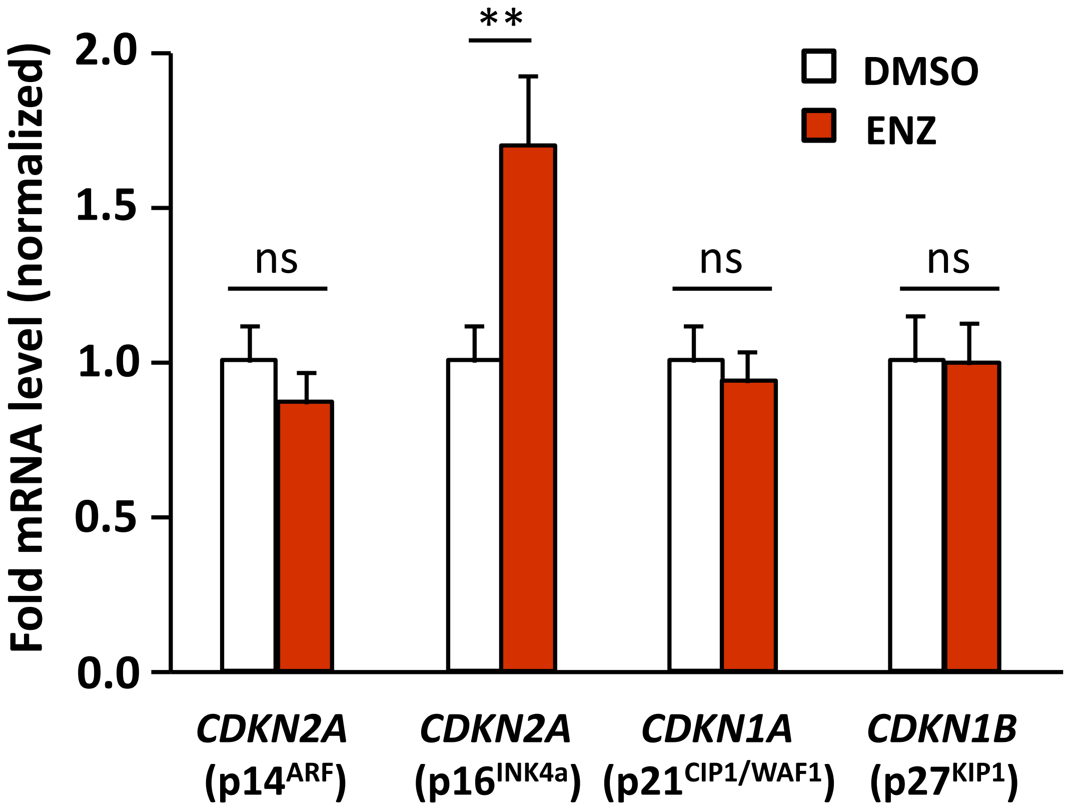

Fig. S1. Enzalutamide treatment significantly induces *CDKN2A* ($p16^{INK4a}$) expression. LNCaP cells were treated 72 h with 10 µM ENZ or 0.1% DMSO. After that, the RNA was extracted and analyzed by qRT-PCR. The mRNA expressions of *CDKN2A* ($p14^{ARF}$), *CDKN2A* ($p16^{INK4a}$), *CDKN1A* ($p21^{CIP1/WAF1}$), and *CDKN1B* ($p27^{KIP1}$) were normalized to the mRNA level of *TBP* as house keeping gene. Bar graphs are shown as mean ± standard deviation (n = 4).

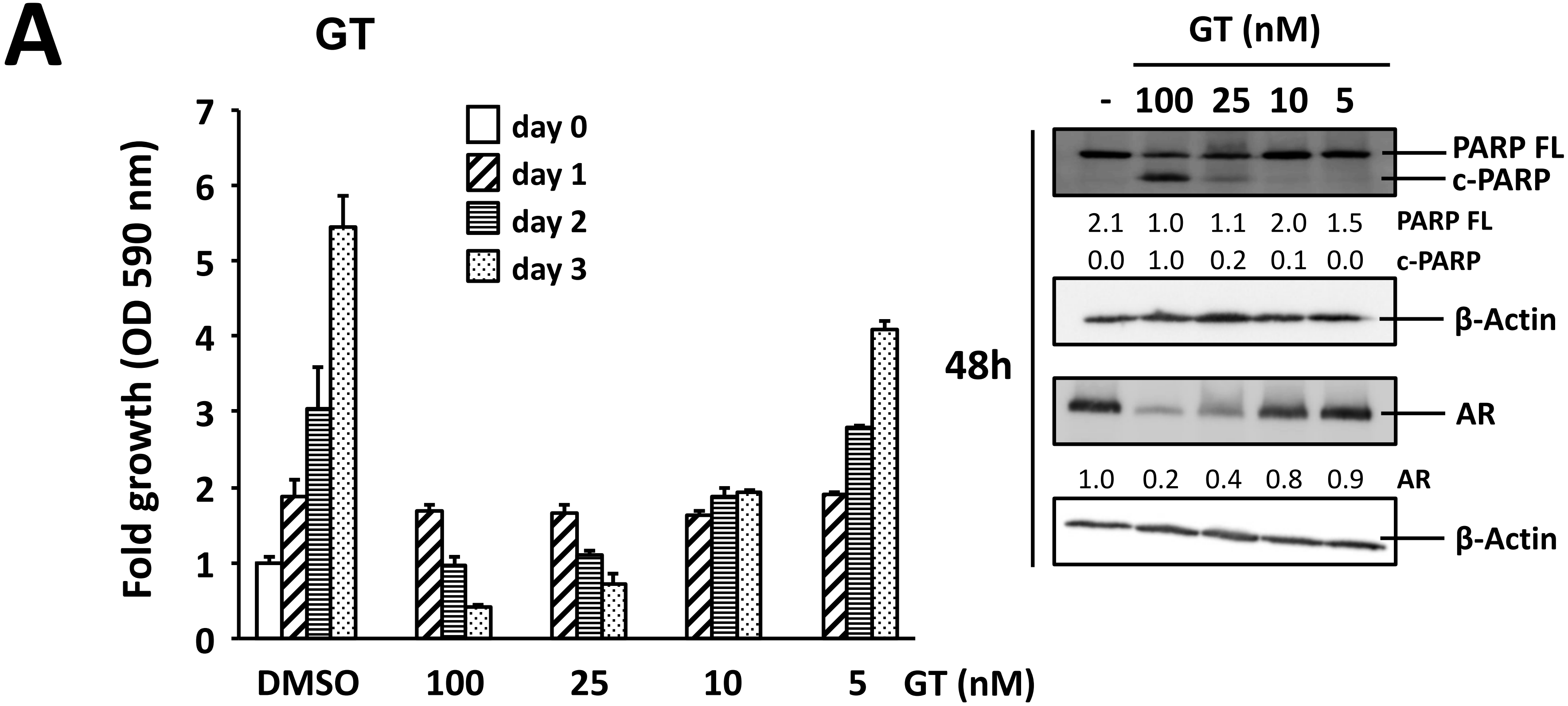

Fig. S2. GT, ABT263, and MK2206 inhibit LNCaP cells proliferation through apoptosis induction in a concentration dependent manner. LNCaP cells were treated 72 h with indicated concentration of A) GT, B) ABT263, C) MK2206, or 0.1% DMSO. Left panel: Growth of LNCaP cells with indicated treatments and time points analysed by crystal violet staining and OD 590 nm measurement. Values obtained from day 0 were set arbitrarily as 1. Bar graphs are shown as mean ± standard deviation (n = 2). Right panel: The protein was extracted and detection of full-length PARP (PARP FL), cleaved PARP (c-PARP), and androgen receptor (AR) was performed by Western blotting. β-Actin served as loading control. Reduction of growth and induction of c-PARP are detected after 48 h treatment with GT and after 24 h with either ABT263 or MK2206.

B

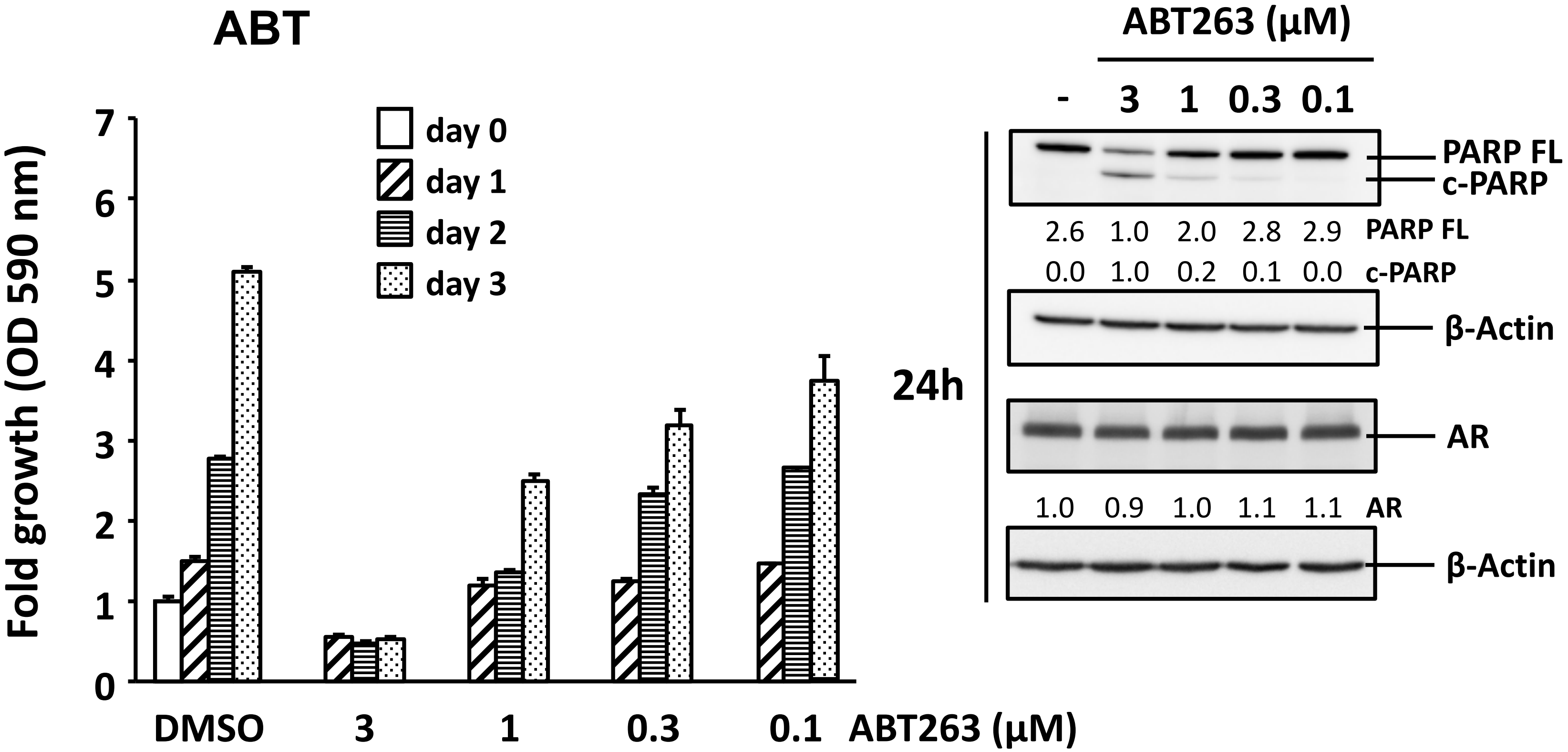
ABT
Fold growth (OD 590 nm)
0
1
2
3
4
5
6
7
day 0
day 1
day 2
day 3
DMSO
3
1
0.3
0.1
ABT263 (μM)
ABT263 (μM)
-
3
1
0.3
0.1
PARP FL
c-PARP
2.6 1.0 2.0 2.8 2.9 PARP FL
0.0 1.0 0.2 0.1 0.0 c-PARP
β-Actin
24h
AR
1.0 0.9 1.0 1.1 1.1 AR
β-Actin

C

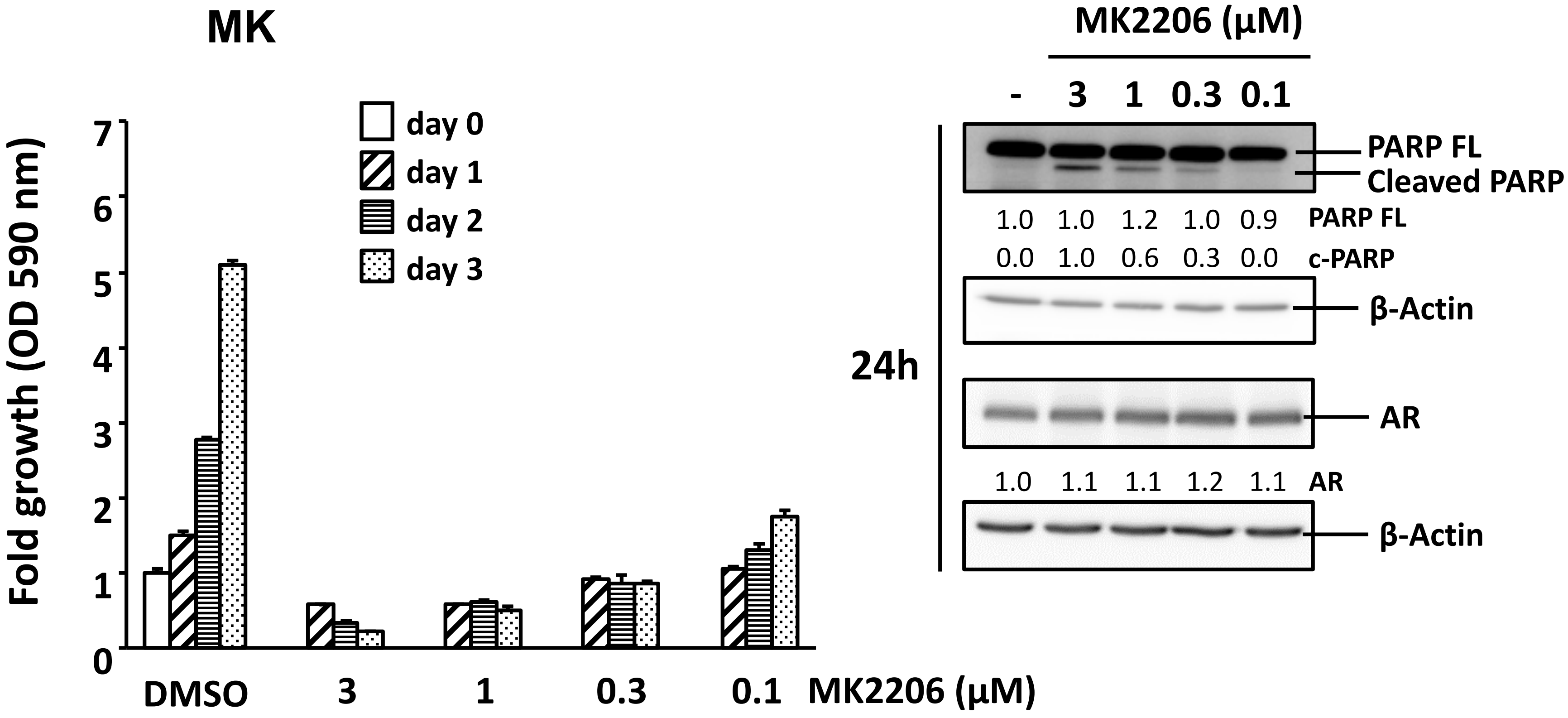
MK
day 0
day 1
day 2
day 3
Fold growth (OD 590 nm)
0
1
2
3
4
5
6
7
DMSO
3
1
0.3
0.1
MK2206 (μM)
MK2206 (μM)
- 3 1 0.3 0.1
PARP FL
Cleaved PARP
1.0 1.0 1.2 1.0 0.9 PARP FL
0.0 1.0 0.6 0.3 0.0 c-PARP
β-Actin
24h
AR
1.0 1.1 1.1 1.2 1.1 AR
β-Actin

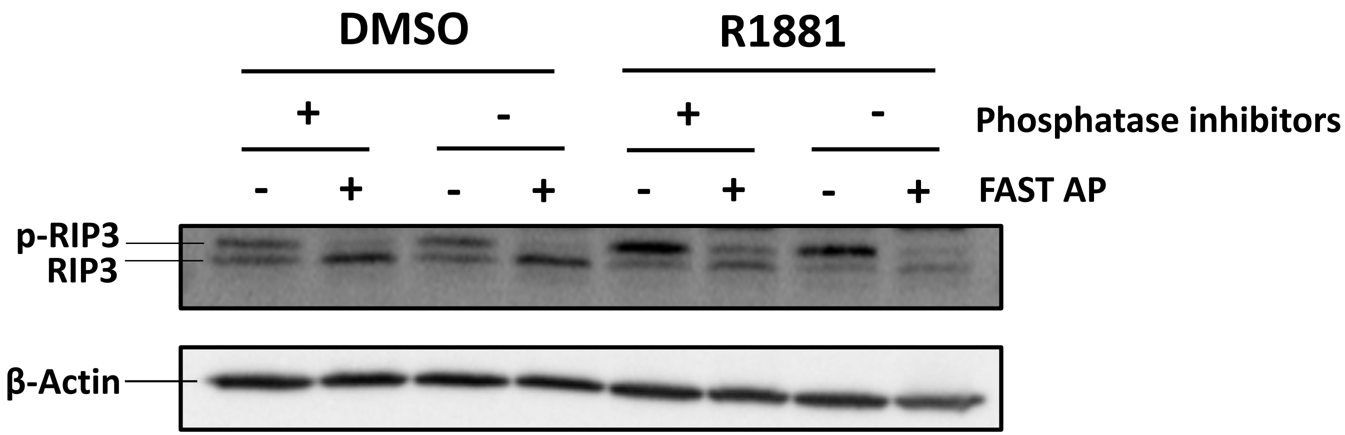

Fig. S3. p-RIP3 is dephosphorylated by alkaline phosphatase (FAST AP). LNCaP cells were treated for 72 h with 1 nM R1881 or 0.1% DMSO as control. After that, the protein was extracted with or without phosphatase inhibitor. The protein extracts were treated with or without alkaline phosphatase (FAST AP) for 3 h at 37°C. Western blotting and detection of phosphorylated RIP3 (p-RIP3) and RIP3 were performed with a specific antibody against RIP3. β-Actin served as loading control.

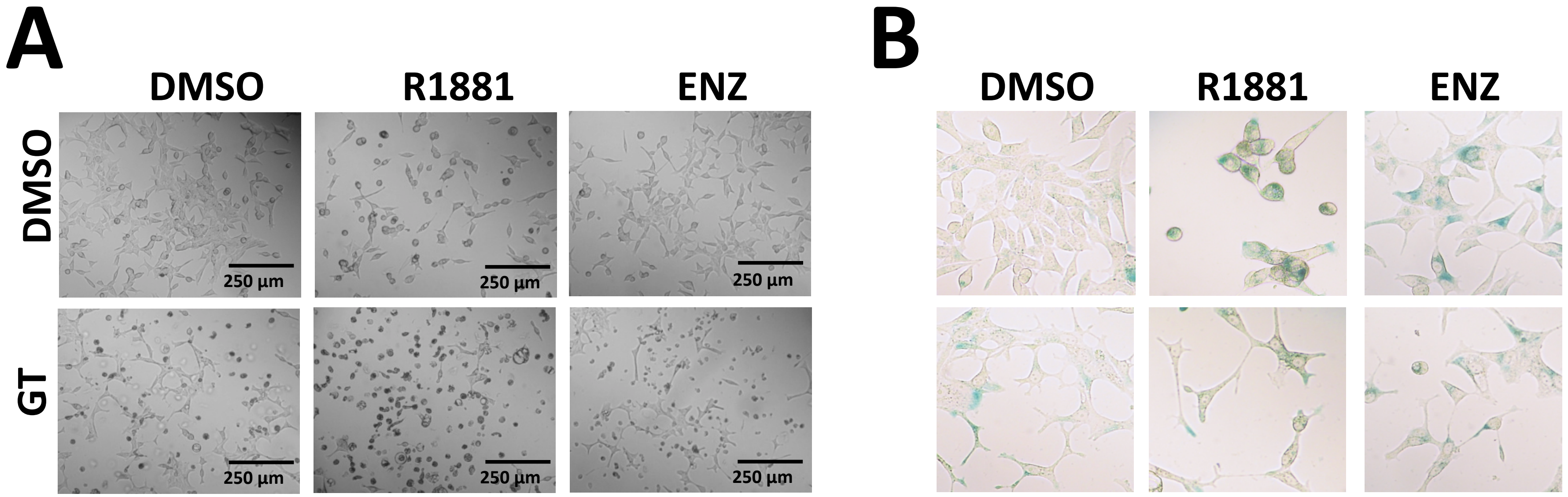

Fig. S4. GT enhances detachment of cells after androgen-induced cellular senescence. LNCaP cells were treated for 72 h with 1 nM R1881, 10 µM ENZ, or 0.1% DMSO as control. After that, the AR ligands were removed. Fresh medium with 0.1% DMSO or 25 nM GT was added and further incubated for additional 48 h. A) Representative pictures of detaching cells under light microscope by particular treatments. B) Representative pictures of SA-β-Gal staining under light microscope.

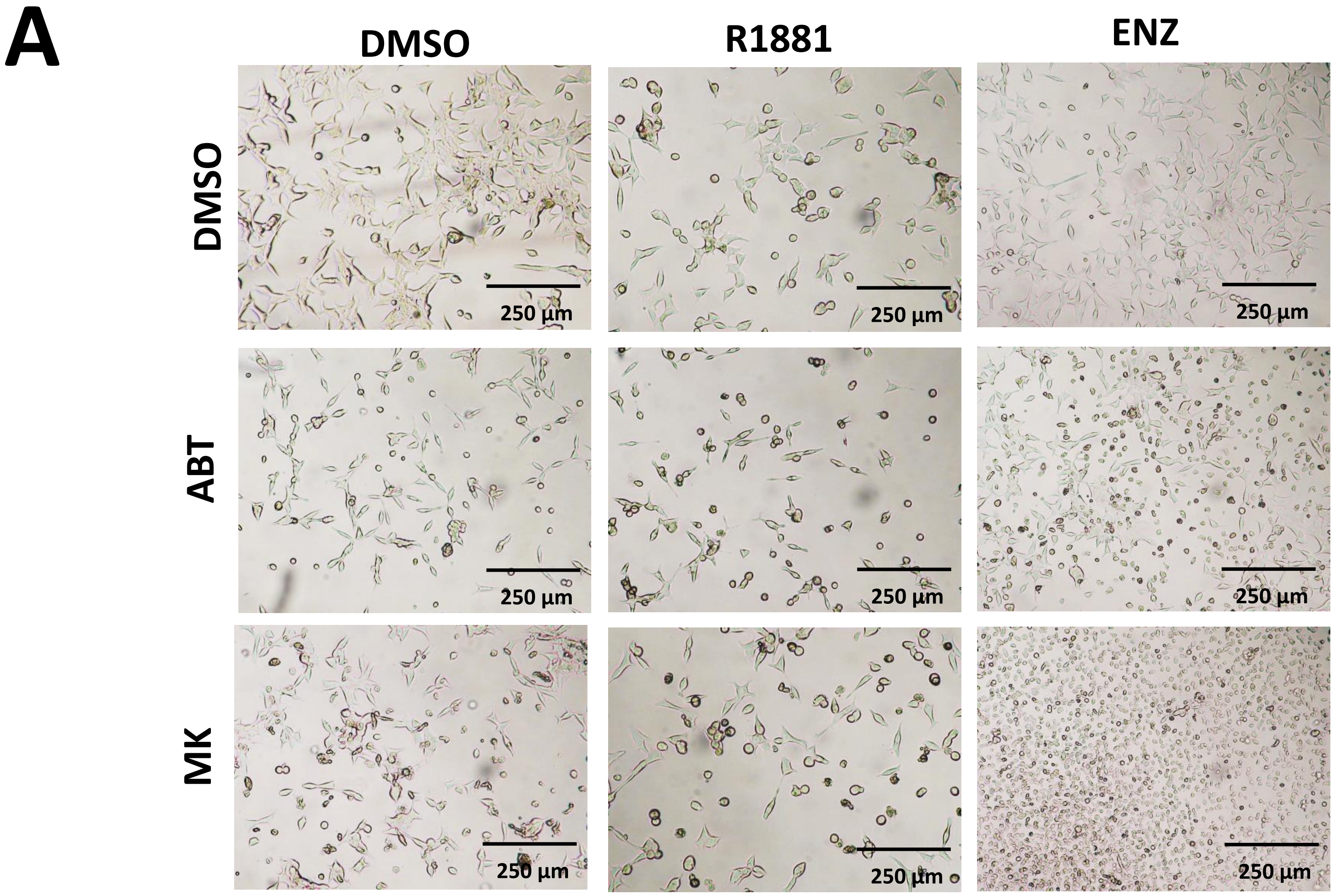

Fig. S5. ABT263 and MK2206 induce LNCaP cell detachment. LNCaP cells were treated for 72 h with 1 nM R1881, 10 µM ENZ, or 0.1% DMSO as control. After that, the AR ligands were removed. Fresh medium with 1 µM ABT263, 1 µM MK2206, or 0.1% DMSO was added, and further incubated for additional 72 h. A) Representative pictures of detaching cells under light microscope by indicated conditions. B) Representative pictures of SA-β-Gal staining under light microscope. C) Percentage of SA-β-Gal positive stained cells. Bar graphs are shown as mean ± SEM (n = 3)

B

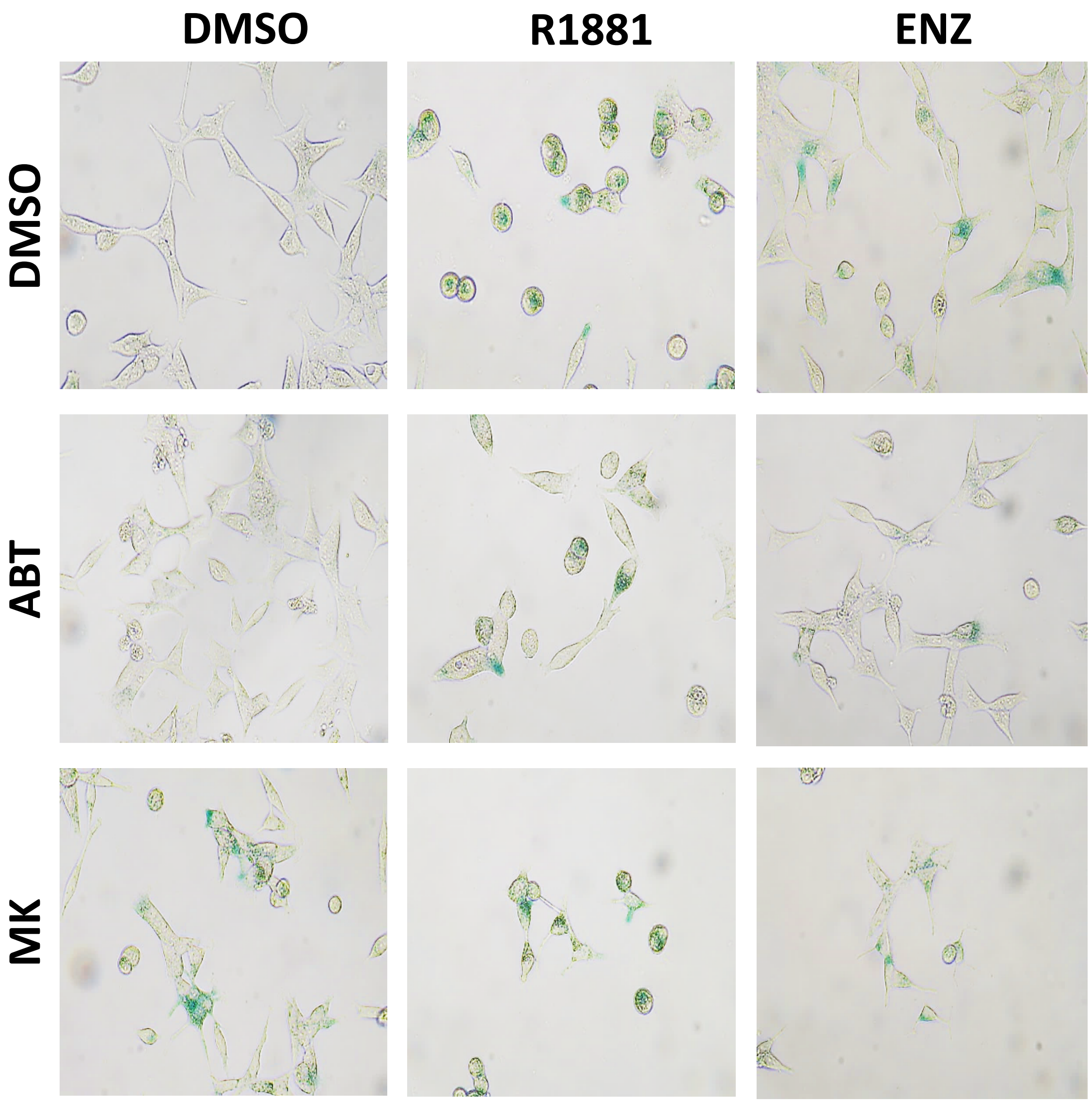

C

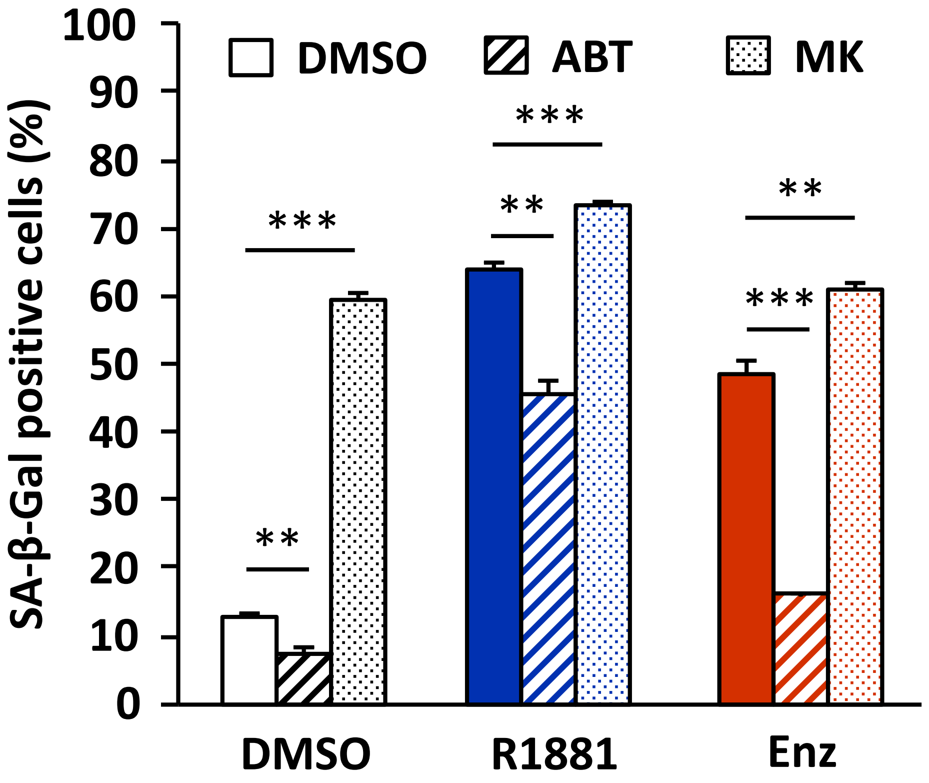

DMSO
ABT
MK
SA-β-Gal positive cells (%)
100
90
80
70
60
50
40
30
20
10
0
***
**
***
**
**
***
DMSO
R1881
Enz

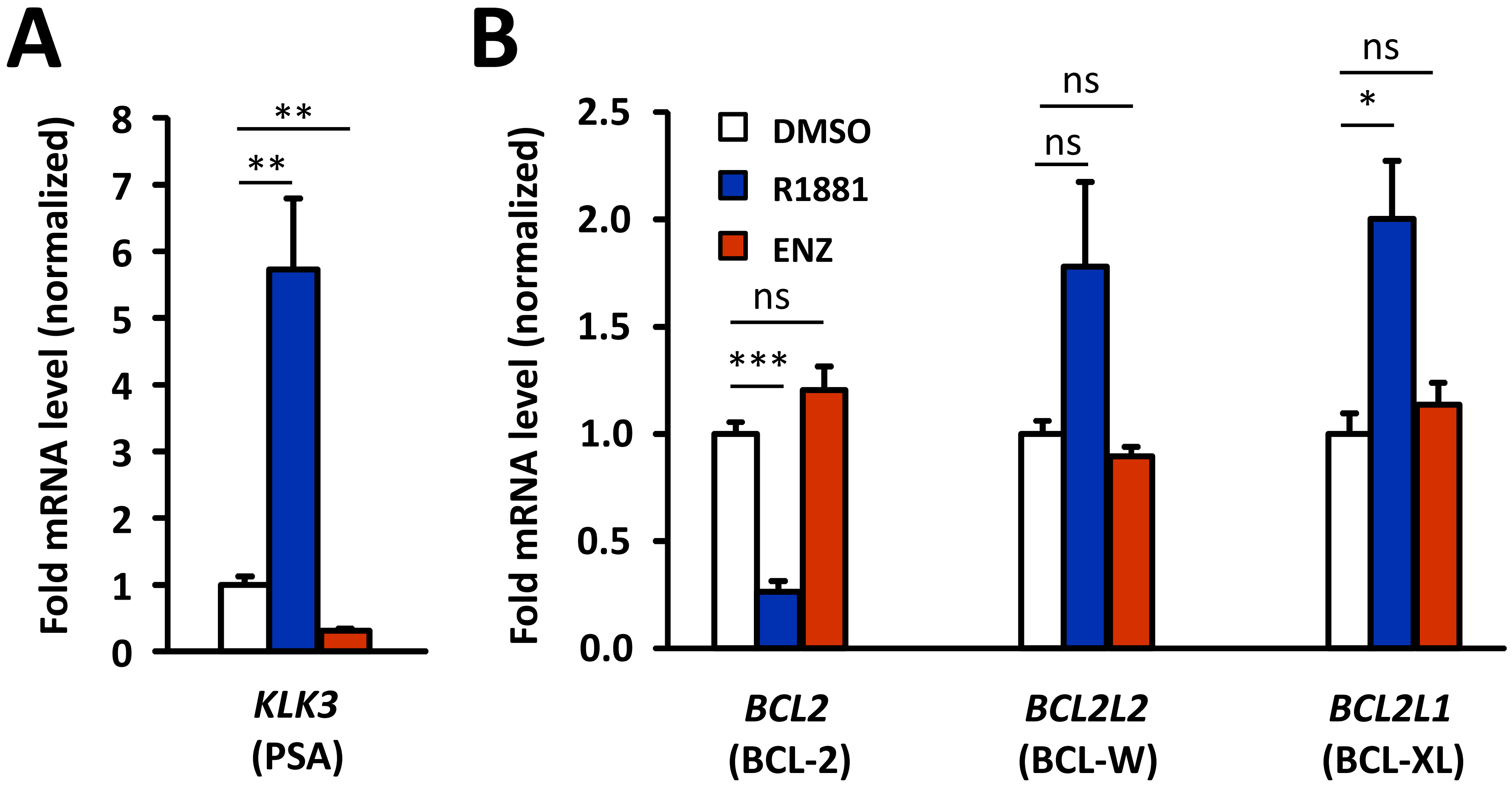

Fig. S6. Androgen regulates Bcl-2 family members transcription levels. LNCaP cells were treated for 72 h with 1 nM R1881, 10 µM ENZ, or 0.1% DMSO as control. After that, the RNA was extracted and analyzed by qRT-PCR. The mRNA expressions of A) *KLK3* (PSA), B) *BCL2* (BCL-2), *BCL2L2* (BCL-W), and *BCL2L1* (BCL-XL) were normalized to the mRNA level of *TBP* as house keeping gene. Bar graphs are shown as mean ± standard deviation (n = 4).
